# Who are they? Identifying risk factors of loss to follow up among HIV+ patients on care and treatment in Dar es Salaam

**DOI:** 10.1186/1471-2334-14-S2-P78

**Published:** 2014-05-23

**Authors:** Lameck C Machumi, Expeditho Mtisi, Irene Andrew, David Sando, Humphrey Mkali, Enju Liu, Ellen Hertzmark, Donna Spiegelman, Wafaie Fawzi, Guerino Chalamilla

**Affiliations:** 1 Management and Development for Health, Dar es Salaam, Tanzania

## Introduction

Loss to follow up (LTFU) is a challenge in care and treatment programs in Sub Saharan Africa, Tanzania included. We analyzed risk factors LTFU among HIV+ patients receiving care and treatment in Dar es Salaam, Tanzania in order to inform strategies to retain patients in care and treatment services.

## Materials and methods

We conducted prospective cohort study of patients enrolled in care and treatment facilities in Dar es Salaam from October 2004 to September 2011. LTFU was defined as missing clinic visit for 90 and 180 consecutive days after the last scheduled appointment date among patients on ART and care and monitoring respectively. For univariate and multivariate analysis, Cox proportional hazard regression model was employed to identify the risk factors.

## Results

Among 85,608 patients followed, 77% were on antiretroviral therapy (ART). The median age of participants was 34 (interquartile range, IQR: 29- 41 years) and their median CD4+ cell count was 206 cells/ L (IQR: 84-378 cells/µL). For those on ART, it was found that patients aged ≥50 years and those with CD4+ cell count <100 cells/ L had an independent significantly increased risk of loss to follow up (RR: 1.11, 95% CI 1.03 – 1.19, p< 0.0001 and RR: 1.22, 95% CI 1.10 – 1.24, p=0.01 respectively). Among patients on care and monitoring male patients, patients with advanced disease and lower CD4 cell count were found to have significantly increased risk with (RR: 1.06, 95% CI 1.01 – 1.14, p< 0.04), (RR: 1.26, 95% CI 1.14 – 1.39, p< 0.0001) and (RR: 2.10, 95% CI 2.07 – 2.22, p< 0.0001) respectively. Patients on care and monitoring were significantly more likely to be LTFU (45%) compared to patients on ART(21%).

## Conclusions

Determining risk of LTFU at enrollment and initiation of ART and focused tracking are crucial to improve retention rates for patients on ART and care and monitoring. Strengthening access and immediate tracking of patients on care and monitoring is recommended to improve patient outcomes, detection and documenting deaths.

